# Supplementary material for: Patterns of Intron Gain and Loss in Fungi
Source: PLoS Biol. 2004 Nov 30;2(12):e422. doi: 10.1371/journal.pbio.0020422 (PMC532390; doi:10.1371/journal.pbio.0020422)
Supplement: Table S1 — Also available at http://genes.mit.edu/NielsenEtAl/. (4.3 MB ZIP). [file pbio.0020422.st001.zip › NielsenEtAl/html/329.html]

AN2062.1.NCU03982.1.MG02503.1.FG09471.1


```
 CLUSTAL W (1.82) Multiple Sequence Alignments - Introns Inserted


Sequence 1: NCU03982.1	661 aa
Sequence 2: MG02503.1	656 aa
Sequence 3: FG09471.1	682 aa
Sequence 4: AN2062.1	674 aa
Alignment Length: 693 aa
Number Identitical Residues: 472 aa
Alignment Score (without introns) 19355


MG02503.1 	-------------MAIGLGMVFWLCLLFSPLAFVKTANAQD----ADSYGTVIGI0DLGT
NCU03982.1	MEHRTR------SWALGLSILGFFALLFS-AGFVQQAHAND----TEAMGTVIGI0DLGT
FG09471.1 	MARSRS------SMALGLGLLCWIALLFSPLAFVQTVQADD----VDSYGTVIGI0DLGT
AN2062.1  	MARPSSGRGVAKPFATWTTIFYLLLVFIAPLAFFGSAHAQEDDSVQENYGTVIGI~DLGT
          	      . . :.. *    :.  : ::::. .*.  .:*::..:  :  ****** ****

MG02503.1 	TYS~CVGVMQKGKVEILVNDQGNRITPSYVAFTDDERLVGDAAKNQAASNPFRTIFDVK2
NCU03982.1	TYS2CVGVMQKGKVEILVNDQGNRITPSYVAFTDEERLVGDAAKNQAAANPHRTIFDIK~
FG09471.1 	TYS~CVGVMQKGKVEILVNDQGNRITPSYVAFTEDERLVGDAAKNQAAANPTNTIYDIK2
AN2062.1  	TYS2CVGVMQNGKVEILVNDQGNRITPSYVAFTDEERLVGDAAKNQYAANPKRTIFDIK2
          	*** ******:**********************::*********** *:** .**:*:* 

MG02503.1 	RLIGRKFTDKDVQQDIKHFPYK~VVNKGGKPVVSVDVKGETKQFTPEEVSAMILGK~MKE
NCU03982.1	RLIGRKFSDKDVQNDIKHFPYK2VVSKDDKPVVKVEVKGEEKTFTPEEISAMILGK0MKE
FG09471.1 	RLIGRKYSEKTLQGDIKHFPFK~VVNRDDRPVVQVEVDGAKKQFTPEEISAMVLGK~MKE
AN2062.1  	RLIGRKYDEADVQRDIKNYPFK~VVNKDGKPNVKVDVNQTPKTLSPEEVSAMVLGK~MKE
          	******: :  :* ***::*:* **.:..:* *.*:*.   * ::***:***:*** ***

MG02503.1 	VAEAYLGKKVTHAVVTVPAYFN~DNQRQATKDAGVIAGLNVLRIVNEPTAAALAYGLDKT
NCU03982.1	TAEGYLGKKVTHAVVTVPAYFN~DNQRQATKDAGMIAGLNVLRIVNEPTAAAIAYGLDKT
FG09471.1 	VAEGYLGKKVTHAVVTVPAYFN0DNQRQATKDAGIIAGLNVLRIVNEPTAAAIAYGLDKN
AN2062.1  	IAEGYLGKKVTHAVVTVPAYFN~DAQRQATKDAGTIAGLNVLRVVNEPTAAAIAYGLDKG
          	 **.****************** * ********* ********:********:****** 

MG02503.1 	GEENQIIVYDLGGGTFDVSLLSIDNGVFEVLATAGDTHLGGEDFDQRIINHFVKMHNKKH
NCU03982.1	GEERQIIVYDLGGGTFDVSLLSIEQGVFEVLSTAGDTHLGGEDFDQRIINHFAKLFNKKH
FG09471.1 	DGERQIIVYDLGGGTFDVSLLSIDDGIFEVLATAGDTHLGGEDFDQRVINYFAKQYNQKN
AN2062.1  	SEERQVIVYDLGGGTFDVSLLSIDDGVFEVLATAGDVHLGGEDFDHRVMDHFVKQYNKKN
          	. *.*:*****************::*:****:****.********:*::::*.* .*:*:

MG02503.1 	NVDVTKDAKAMGKLKREAEKAKRTLSSQKSTRIEIESFFDGKDFSETMTQAKFEELNIDL
NCU03982.1	GVDVTKDAKAMGKLKREAEKAKRTLSSQMSTRIEIEAFYDGKDFSETLTRAKFEELNNDL
FG09471.1 	NVDITKDLKAMGKLKREAEKAKRTLSSQKSTRIEIEAFHAGKDFSETLTQAKFEELNIDL
AN2062.1  	NVDVTKDLKAMGKLKREVEKAKRTLSSQMSTRIEIEAFHNGQDFSETLTRAKFEELNMDL
          	.**:*** *********.********** *******:*. *:*****:*:******* **

MG02503.1 	FKKTLKPVEQVLKDAKLKKEDINDIVLVGGSTRIPKVQALIKEYFNGKNPSKGINPDEAV
NCU03982.1	FKKTLKPVEQVLKDAKVSKSEIDDIVLVGGSTRIPKVQALIEEFFNGKKASKGINPDEAV
FG09471.1 	FKKTMKPVEQVLKDAKLKKSEIDDIVLVGGSTRIPKVQQLIEDFFN-KKASKGINPDEAV
AN2062.1  	FKKTLKPVEQVLKDAKVSKADIDDIVLVGGSTRIPKVQAMLEEFFDGKKASKGINPDEAV
          	****:***********:.* :*:*************** :::::*:.*:.**********

MG02503.1 	AFGAAVQAGVLSGEEGTGDVVLMDVNPLTLGIETTGGVMTKLIPRNTPIPTRKSQIFSTA
NCU03982.1	AFGAAVQAGVLSGEEGTEDIVLMDVNPLTLGIETTGGVMTKLIPRNTPIPTRKSQIFSTA
FG09471.1 	AFGAAVQAGVLSGEEGTSGVVLMDVNPLTLGIETTGGVMTKLIPRNTAIPTRKSQIFSTA
AN2062.1  	AFGAAVQGGVLSGVEGTESIVLMDVNPLTLGIETTGGVMTKLIPRNTVIPTRKSQIFSTA
          	*******.***** *** .:*************************** ************

MG02503.1 	ADNQPVVLIQVF~EGERTMTKDNNLLGKFELTGIPPAPRGQPQIEVSFELDANGILKVSA
NCU03982.1	ADNQPVVLIQVY~EGERSMTKDNNLLGKFELTGIPPAPRGVPQIEVSFELDANGILKVSA
FG09471.1 	ADNQPVVLIQVF~EGERSLTKDNNILGKFELTGIPPAPRGVPQIEVSFELDANGILKVSA
AN2062.1  	ADNQPTVLIQVY1EGERTLTKANNLLGKFELTGIPPAPRGVPQIEVSFDLDANGILKVGA
          	*****.*****: ****::** **:*************** *******:*********.*

MG02503.1 	HDKGTGNTEQITITNDKGRLTPEEIDRMVTEAEKFAEEDKATRERIEARNGLENYAFSLK
NCU03982.1	HDKGTGKAESITITNDKGRLTQEEIDRMVAEAEKYAEEDKATRERIEARNGLENYAFSLK
FG09471.1 	HDKGTGKQESITITNDKGRLTPEEIERMVAEAEKYAEEDKATRERIEARNGLENYAFSLK
AN2062.1  	SDKGTGKAESITITNDKGRLTQEEIDRMVAEAEEFAEEDRAIKAKIEARNSLENYAFSLK
          	 *****: *.*********** ***:***:***::****:* : :*****.*********

MG02503.1 	NQVKDEEGLGGKIDESDKE-------------------A0LLDAIKEATEWLEENG~ATA
NCU03982.1	NQVNDEDGLGGKIDEEDKE-------------------T~ILDAVKEAQDWLEENA1ATA
FG09471.1 	NQVNDEEGLGGKIDEEDKETVSSHTTLNNKSKTHANKSQ~LLDAVKETNEWLDEHG~ADA
AN2062.1  	NQVNDENGLGGQIDEDDKQ-------------------T~ILDAVKEVNDWLEENG~ASA
          	***:**:****:***.**:                     :***:**. :**:*:. * *

MG02503.1 	EAADFEEQKEKLSGVAYPITSKLYS-GGAGGDSDEPRSHDEL
NCU03982.1	SAEDFDEQKEKLSNVAYPITSKLYS-QGGAGD-DEPAGHDEL
FG09471.1 	TAEDFEEQKEKLSNVAYPITSKMYQGAGGAGGEQDDNIHDEL
AN2062.1  	TTEDFEEQKEQLSNVAYPITSKLYGSAPPTEEDDDEYGHDEL
          	 : **:****:**.********:* .      .::   ****
```
